# Current Clinical Perspectives of Biomarkers in Respiratory Diseases: A Narrative Review

**DOI:** 10.3390/jcm15145708

**Published:** 2026-07-21

**Authors:** Swathi Gurajala, Shoug Yousif Al Humoud, Ghada Fouad Al Yousif, Rana Ali Alameri, Gayathri Pandurangam, Aya Khalid Ali Fayyomi, Sally Abed, Nada Sami Sardidi, Mashael Mamdouh Alrayes, Tarfah Ahmed Alsabhan, Sarah Hassan Alajmi, Anfal Alfaraj, Nada Al Ghannam

**Affiliations:** 1 Department of Respiratory Care, College of Applied Medical Sciences in Jubail, Imam Abdulrahman Bin Faisal University, Al Jubail 35816, Saudi Arabia; gppandurangam@iau.edu.sa (G.P.); akfayyomi@iau.edu.sa (A.K.A.F.); syabed@iau.edu.sa (S.A.); nssardidi@iau.edu.sa (N.S.S.); mmalrayes@iau.edu.sa (M.M.A.); taalsabhan@iau.edu.sa (T.A.A.); shalajmi@iau.edu.sa (S.H.A.); aamalfaraj@iau.edu.sa (A.A.); nsghannam@iau.edu.sa (N.A.G.); 2 Department of Respiratory Care, Imam Abdulrahman Bin Faisal University, Dammam 34212, Saudi Arabia; salhumoud@iau.edu.sa; 3 Department of Family and Community Medicine, College of Medicine, Imam Abdulrahman Bin Faisal University, Dammam 34212, Saudi Arabia; gfalyousif@iau.edu.sa; 4 Fundamentals of Nursing Department, College of Nursing, Imam Abdulrahman Bin Faisal University, Dammam 34212, Saudi Arabia; raalamri@iau.edu.sa

**Keywords:** respiratory diseases, biomarkers, non-invasive approaches, novel diagnostic tests, precision medicine, personalized therapy, disease phenotyping, diagnostic tools, functional biomarkers, imaging biomarkers, molecular biomarkers, artificial intelligence

## Abstract

Respiratory medicine is transitioning from symptom-driven, standardized care to a more precise, patient-specific approach guided by molecular profiling. This evolution is being enabled by advances in liquid biopsy, multiomics, and artificial intelligence (AI) analytics. Fractional exhaled nitric oxide (FeNO) and blood eosinophils, the two commonly used markers in asthma, are now being joined by more precise airway markers such as galectin-10, which could aid clinicians in making more informed decisions for biological treatments. In chronic obstructive pulmonary disease (COPD) similar progress is underway, with treatment now emphasizing inflammation endotypes, especially eosinophilic patterns, to direct therapeutic choices. Alongside these developments, routine blood-based ratios (e.g., platelet-to-lymphocyte and neutrophil-to-lymphocyte) are being explored as predictors of exacerbation risk, and forced oscillation testing (FOT) is proving useful for picking up early disease shifts. In more severe conditions, biomarkers are linked to an early and better prognosis, enabling timely intervention. Markers like Matrix metalloproteinase-7 (MMP-7) and CC chemokine ligand 18 (CCL18) have proven to be reliable indicators of mortality and disease progression in idiopathic pulmonary fibrosis. Meanwhile, in lung cancer, liquid biopsies, especially those measuring circulating tumor DNA and micro-RNA (miRNA) panels, are enhancing screening accuracy while helping to cut down on the high false-positive rates seen with low-dose computerised tomography (CT). Other respiratory conditions such as bronchiectasis, pulmonary embolism, pneumonia, and acute respiratory distress syndrome (ARDS) are also benefiting from biomarker advances. At the same time there is a growing push to standardize how these biomarkers are measured. AI-based clinical decision support systems are also playing an increasingly important role in the translation of all these complicated data into actionable clinical insights. Together these developments pave the way for improved respiratory care that is precise and responsive to individual patient needs.

## 1. Introduction

Chronic respiratory diseases (CRDs), primarily chronic obstructive pulmonary disease (COPD) and asthma, affect between 454 and 545 million people worldwide and result in nearly 4 million deaths annually. While age-standardized rates for prevalence and mortality have actually decreased, the absolute number of cases has surged by nearly 40% since 1990. This absolute increase is driven largely by population growth and an aging global demographic [1]. CRDs account for over 103 million disability-adjusted life-years (DALYs) globally, representing a massive clinical burden heavily influenced by ubiquitous environmental, occupational, and behavioral exposures. Risk factors vary significantly by demographic; while smoking remains the leading driver of disability for men globally, women in certain regions are disproportionately impacted by alternative inhalational risks, such as ambient particulate matter and household air pollution from solid fuels [2].

Traditionally, the diagnosis and prognostic evaluation of chronic respiratory conditions such as asthma, COPD, idiopathic pulmonary fibrosis (IPF), and lung cancer rely on the clinical presentation, radiological investigations, and pulmonary function tests [3]. These modalities have certain limitations in identifying the multifactorial and complex immunopathogenesis mechanisms underlying airway and parenchymal diseases [4]. This diagnostic uncertainty often results in a “one-size-fits-all” approach, leading to an overprescription of systemic corticosteroids, failure to stop disease progression at early, modifiable stages, and eventually suboptimal patient outcomes [5].

Recently, the medical field has been transformed by the widespread integration of molecular biomarkers. Biomarkers are defined as quantifiable parameters that serve as objective indicators of normal biological processes, pathogenic mechanisms, or pharmacological responses in humans [6]. The rapid evolution of multiomics technologies, combined with advancements in high-throughput single-cell and spatial sequencing, has led to the discovery of organ-specific molecular signatures [7]. These molecular insights help to classify patients into specific biological endotypes to guide the application of highly targeted therapies, such as monoclonal antibodies and small-molecule inhibitors [8].

The detection of biomarkers has expanded beyond traditional invasive tissue biopsies to minimal/non-invasive methods such as liquid biopsies (blood, urine, cerebrospinal fluid) and exhaled breath profiling (breath omics), thereby reducing patient morbidity and facilitating disease monitoring [9]. The data generated by these advanced assays require robust computational frameworks; consequently, artificial intelligence (AI) and machine learning (ML) are now integral parts of the validation and clinical deployment of biomarker panels [10]. The current review consolidates the available literature on the diagnostic, prognostic, and therapeutic applications of biomarkers in major respiratory diseases, with emphasis on their incorporation into contemporary clinical guidelines and translational challenges.

## 2. Biomarkers in Obstructive Airway Diseases

### 2.1. Asthma

Asthma is a heterogeneous clinical syndrome driven by distinct, overlapping inflammatory pathways. The transition from symptom control to targeted disease modification depends on the identification of specific molecular phenotypes. Consequently, the identification and continuous monitoring of biomarkers have become central in asthma management in guiding the selection of advanced biologic therapies [5].

#### 2.1.1. Type-2 (T2) Inflammatory Biomarkers

The 2026 update to the Global Initiative for Asthma (GINA) strategy formally lists the diagnostic and prognostic role of established type 2 (T2) biomarkers: peripheral blood eosinophils, fractional exhaled nitric oxide (FeNO), serum immunoglobulin E (IgE) and sensitization to an aeroallergen. These markers are critical for distinguishing T2-high asthma, which is characterized by intrinsic allergic or eosinophilic pathways, from T2-low asthma [11].

The peripheral blood eosinophil count is the most accessible surrogate for eosinophilic airway inflammation. Elevated peripheral eosinophils are associated with an increased risk of severe asthma exacerbation, failure to achieve symptom control, fixed airflow obstruction, and accelerated decline in pulmonary function. Importantly, landmark retrospective data demonstrate that concomitant systemic and bronchial eosinophilic inflammation significantly contributes to poor asthma control, underscoring the clinical necessity of dual-compartment biomarker assessment [12]. Furthermore, combining blood eosinophilia with fractional exhaled nitric oxide (FeNO) provides a simple, highly predictive index that is strongly associated with greater asthma exacerbation rates [13]. More importantly, the specific quantification of blood eosinophils (typically categorized by thresholds such as ≥150 cells/µL (lower threshold) or ≥300 cells/µL (higher threshold)) serves as an important predictive biomarker for therapeutic responsiveness to newer T2-targeted biologics, which include anti-IL-5 agents (mepolizumab, reslizumab), anti-IL-5Rα agents (benralizumab), and anti-IL-4Rα agents (dupilumab) [11].

Fractional exhaled nitric oxide (FeNO), which acts primarily as a surrogate for interleukin-13 (IL-13)-driven processes, provides complementary, non-invasive evidence of airway epithelial inflammation [14]. According to the GINA 2026 guidelines, an FeNO level of ≥20 parts per billion (ppb) and elevated eosinophils support the allocation of a patient into a T2-high phenotype, guiding the initiation of specialized biologic treatment pathways [15]. These guidelines also emphasize that lower levels of FeNO or blood eosinophils at a single time point do not definitively rule out asthma or a T2 phenotype [15].

Both biomarkers show physiological variability; blood eosinophils peak and FeNO troughs in the morning. Furthermore, the levels are also influenced by age, sex, and, in the case of FeNO, the specific measurement device and site. The latest GINA 2026 guidelines therefore recommend re-evaluating initially low biomarker values if the clinical context or symptom severity changes [15].

#### 2.1.2. Sputum Diagnostics and Interleukin Profiling

While peripheral blood markers offer logistical convenience, induced sputum analysis provides direct visualization of the cellular and cytokine microenvironments of the lower respiratory tract. Sputum inflammatory cell analysis is the validated gold standard for identifying eosinophilic airway inflammation, which accounts for approximately 50% to 60% of all asthma cases. Quantitative sputum cell counts have proven to be highly effective clinical tools for assessing underlying disease severity and directly monitoring corticosteroid treatment outcomes [16]. Clinically defined as ≥3% of total sputum cells, sputum eosinophilia represents a highly specific marker of T2 airway inflammation and serves as a robust predictive biomarker for both a favorable response to inhaled corticosteroids and the efficacy of advanced asthma biologic therapies [14,16].

In addition to simple cellular enumeration, the quantification of sputum cytokines, specifically interleukins (ILs) 4, 5, 8, 13, and 33, has emerged as a highly promising category of prognostic and monitoring biomarkers. Notably, research indicates that lower levels of sputum IL-13 demonstrate superior diagnostic accuracy compared with both sputum eosinophils and FeNO in identifying well-controlled asthma, suggesting its optimal role as a high-fidelity monitoring biomarker in longitudinal asthma control assessments. Conversely, elevated baseline levels of sputum IL-13 robustly predict a greater therapeutic response to omalizumab [17].

The sputum IL-33 profile offers insights into complex asthmatic sub-phenotypes. High levels of sputum IL-33 are linked to mixed granulocytic asthma, a difficult-to-treat variant. Furthermore, elevated sputum IL-33 levels are observed in asthmatic patients with increased capsaicin cough sensitivity and functional dyspepsia, suggesting a systemic neuroinflammatory link. Additionally, the assessment of sputum neutrophils has significant potential for identifying and classifying T2-low asthma. While this endotype historically lacked targeted interventions, the therapeutic landscape has recently expanded with the introduction of Tezepelumab, an anti-thymic stromal lymphopoietin (TSLP) monoclonal antibody that has demonstrated significant clinical efficacy in reducing exacerbations across both T2-high and T2-low phenotypes [17,18]. Despite its unparalleled diagnostic specificity, routine clinical integration of sputum profiling remains limited because of the need for specialized laboratory expertise, high costs, and time-intensive processing protocols [14].

#### 2.1.3. Serum Galectin-10

Given the limitations of existing markers, highly specific, easily accessible surrogate biomarkers of airway inflammation have been identified. A breakthrough in the past two years has been the extensive validation of galectin-10, the primary constituent protein of Charcot–Leyden crystals. These crystals form rapidly in the airways of patients with severe eosinophilic inflammation, thereby making galectin-10 a direct mechanistic byproduct of eosinophil degranulation and death [14].

Studies have demonstrated a strong association between the levels of galectin-10 in induced sputum and quantitative sputum eosinophil counts, accurately mirroring the gold standard for detecting eosinophilia without requiring complex cellular cytospins [14]. The diagnostic utility of galectin-10, however, extends beyond sputum to peripheral blood. In a 2024 cohort study, median serum Galectin-10 levels were significantly elevated in adult asthmatics (4.7 ng/mL) compared with healthy controls (2.9 ng/mL, *p* < 0.001) [19]. Most importantly, elevated serum Galectin-10 is strongly correlated with severe physiological damage; it is significantly greater in individuals with asthma with fixed, persistent airflow limitation (PAL) (*p* = 0.005) and in those suffering from small airway dysfunction (SAD) (*p* = 0.004) [14].

Phenotypic profiling of patient cohorts utilizes Receiver Operating Characteristic (ROC) curve analysis to stratify patients; serum Galectin-10 successfully discriminated patients with persistent airflow limitation (AUC: 0.719, sensitivity 70%, specificity 66%). Based on these thresholds, the “Galectin-10-high” group presented considerably lower forced expiratory volume in one second (FEV1%) and maximal mid-expiratory flow (MMEF%) values than their “Galectin-10-low” counterparts did, along with higher peripheral CD66+ neutrophil counts and elevated serum periostin. In experimental airway models in which epithelial cells are exposed to house dust mites and eosinophil/neutrophil cocultures, galectin-10 production increases significantly across both eosinophilic and neutrophilic inflammatory conditions, indicating its broad relevance to multiple asthma phenotypes [19].

Additionally, sophisticated liquid biopsy methods that concentrate on serum extracellular vesicles (EVs) have separated galectin-10 from other particular markers including eosinophil peroxidase, major basic protein and arachidonate 15-lipoxygenase. In diagnostic comparative studies, the concentration of galectin-10 encapsulated within these serum EVs proved superior to standard peripheral eosinophil cell counts in detecting airway obstruction and determining diagnostic capability [20].

Therapeutic monitoring also benefits from this marker; longitudinal studies involving patients treated with mepolizumab demonstrated a significant, treatment-induced decrease in both serum and sputum Galectin-10 levels, verifying its potential as a dynamic marker of therapeutic efficacy and disease modification [14].

#### 2.1.4. Additional Investigational Asthma Markers

The pursuit of precision medicine has yielded several other promising candidates aimed at predicting airway remodelling and monitoring subclinical inflammation. Serum matrix metalloproteinase-9 (MMP-9) levels correlate directly with steroid responsiveness in moderate to severe asthmatic patients. Periostin, an inflammatory epithelial-derived protein, is frequently elevated in asthma EBC and serum, indicating active airway remodelling and lung fibrosis. Conversely, early predictive markers such as galectin-3 have shown utility in indicating the modulation of airway remodelling in patients specifically treated with omalizumab. Connective tissue growth factor (CTGF) is currently being mapped in the context of therapies that suppress airway remodelling via anti-TSLP antibodies [14]. In addition to specific proteins, non-invasive matrices such as volatile organic compounds (VOCs) and particles in inhaled air (PexA) are currently being evaluated to extend the diagnostic capabilities of breath omics, capturing broader molecular signatures of airway dysfunction [21].

Urinary leukotriene E4 (LTE_4_) is a stable, non-invasive biomarker used to measure the total production of cysteinyl leukotrienes, which are potent inflammatory mediators involved in airway constriction, mucus production, and allergic responses. Measuring LTE_4_ in urine avoids artifactual changes that can occur in blood draws. Levels increase during acute asthma exacerbations, exposure to environmental triggers, and antigen challenges. It aids in predicting which patients might react favourably to leukotriene receptor antagonists [22].

The cytotoxic enzyme known as eosinophil cationic protein (ECP) is present in white blood cells called eosinophils. It is a vital clinical biomarker that is released during inflammation. It evaluates the intensity and activity of allergies, especially atopic dermatitis, rhinitis and asthma. Serum ECP levels are closely correlated with asthma severity, making it a reliable predictor of exacerbations [23].

#### 2.1.5. Non-T2 Biomarkers

There is a critical lack of validated biomarkers for non-T2 (T2-low) asthma. Non-T2 asthma often presents with sputum neutrophilia and is associated with a poor response to standard inhaled corticosteroids [14]. However, the therapeutic landscape for this challenging endotype is evolving rapidly with the recognition of epithelial-derived alarmins, particularly thymic stromal lymphopoietin (TSLP). Anti-TSLP biologics, such as Tezepelumab, have recently demonstrated significant clinical efficacy in reducing exacerbations across diverse patient populations, notably including those with T2-low phenotypes who previously lacked targeted biological options [24].

YKL-40 is a glycoprotein secreted by immune cells, activated astrocytes, and macrophages. It functions as a key biomarker for systemic inflammation, tissue remodelling, and extracellular matrix degradation, making it a powerful indicator of disease severity and progression. Serum YKL-40 levels in noneosinophilic asthma patients (including those with neutrophilic and paucigranulocytic phenotypes) are approximately 1.5-fold higher than those observed in eosinophilic asthma patients, which may reflect the likely neutrophil-associated origin of YKL-40 [25]. Additionally, a single-nucleotide polymorphism in the *CHI3L1* gene (rs4950928) identified in the American Hutterite population has been linked to increased serum YKL-40 concentrations in individuals with asthma (102.7 ± 2.9 µg/L compared with 87.2 µg/L in healthy controls; *p* = 0.005). However, YKL-40 levels were not correlated with impaired lung function [26].

#### 2.1.6. Epithelial-Derived Alarmins

The airway epithelium acts as an immunological interface, actively responding to environmental insults such as allergens, pollutants, and viruses, by releasing alarmin cytokines, notably thymic stromal lymphopoietin (TSLP), interleukin-33 (IL-33), and interleukin-25 (IL-25). These epithelial-derived alarmins act as upstream master regulators that involve complex downstream inflammatory cascades, driving both traditional T2-skewed eosinophilic inflammation and non-T2 pathophysiological pathways [27]. Because alarmins initiate the inflammatory response prior to its divergence into specific endotypes, their expression serves as a critical indicator of overall airway hyperresponsiveness and disease severity. Furthermore, the central role of these alarmins has established them as highly promising therapeutic targets, as evidenced by the clinical success of anti-TSLP biologics (e.g., Tezepelumab) in reducing exacerbations across diverse asthma phenotypes [28]. Table 1 presents a structured overview of both established and emerging biomarkers used in the diagnosis, phenotyping, and management of asthma.

### 2.2. Chronic Obstructive Pulmonary Disease (COPD)

Chronic obstructive pulmonary disease (COPD) is an accelerating global health burden and currently ranks as the third leading cause of death worldwide [3]. For several years, COPD was defined as irreversible airflow obstruction measured via spirometry, leading to symptomatic therapy [8]. The 2026 latest Global Initiative for Chronic Obstructive Lung Disease (GOLD) report treatment pathway utilizes the ABCD assessment tool, dividing management into Initial Therapy and Follow-Up Adjustments, while aggressively lowering the exacerbation threshold for high-risk status (Group E) and introducing targeted biologic therapies, such as mepolizumab and dupilumab, for specific biomarker-driven phenotypes [29].

#### 2.2.1. Eosinophilic Endotypes of COPD

Classic COPD pathogenesis is driven by tobacco smoke-induced neutrophilic inflammation and macrophage infiltration; a specific subset of COPD patients exhibits T2-like eosinophilic inflammation [8]. Hence, the blood eosinophil count has been identified as a primary prognostic and predictive biomarker in the GOLD guidelines. Initial pharmacologic treatment is guided by symptom severity and exacerbation history, with Group A receiving a single bronchodilator, Group B receiving dual bronchodilation ((Long-Acting Muscarinic Antagonist (LAMA) + Long-acting beta-agonists (LABA)), and the high-risk Group E receiving dual bronchodilation or triple therapy (LAMA + LABA + inhaled corticosteroids (ICS)) if blood eosinophils are ≥300 cells/μL. For patients experiencing persistent symptoms or exacerbations despite maintenance therapy, treatment escalation is dictated by predominant treatable traits; for instance, persistent dyspnea warrants a step-up to dual or triple therapy, or a change in inhaler device. In the context of persistent exacerbations, clinicians should escalate to triple therapy, consider adding roflumilast or macrolides for patients with low eosinophils, and introduce biologics for those with chronic bronchitis and continued exacerbations despite maximized triple therapy. Alongside these pharmacological strategies, the guidelines mandate core non-pharmacological interventions, including smoking cessation, pulmonary rehabilitation, verification of inhaler technique prior to therapy escalation, and updated vaccinations for influenza, pneumococcal disease, and respiratory syncytial virus (RSV) [29]. This biomarker-driven decision tree is vital, as administering ICS to COPD patients with low blood eosinophil counts provides less therapeutic benefit and paradoxically exposes them to a significant risk of developing pneumonia [30].

The identification of this treatable trait has ushered in the first wave of targeted biologic therapies for COPD. FDA-approved biologics, such as mepolizumab (anti-IL-5) and dupilumab (anti-IL-4Rα), have proven capable of significantly reducing acute exacerbations, limiting long-term systemic steroid exposure, and rewriting airway disease management by bringing asthma-like precision to COPD care [8]. Concurrently, fractional exhaled nitric oxide (FeNO) is being increasingly studied as a non-invasive adjunct to identify and monitor these eosinophilic COPD endotypes [30].

#### 2.2.2. Systemic Inflammatory Markers

Acute exacerbation of COPD (AECOPD) is a life-threatening clinical event that increases systemic inflammatory responses, accelerates lung function loss, triggers acute respiratory failure, and significantly increases both cardiovascular complication rates and overall mortality risk [31]. Predicting which patients are prone to the “frequent exacerbator” phenotype is a critical objective for resource management and tertiary prevention [30].

Traditionally, acute-phase reactants such as elevated levels of C-reactive protein (CRP) and fibrinogen are consistently associated with increased exacerbation risk and severe disease progression [3]. Procalcitonin measurement is routinely deployed to differentiate bacterial infections from viral exacerbations and serves as a critical biomarker for guiding targeted antimicrobial therapy [30]. A prospective, multicentre study involving intensive care unit (ICU) patients with H1N1 influenza reported that procalcitonin demonstrated a 94% negative predictive value for ruling out bacterial coinfection [32]. Furthermore, deep cytokine profiling revealed that the levels of interleukins 6, 8, 33 (IL-6, IL-8, and IL-33) are consistently elevated in individuals with frequent exacerbations, reflecting sustained neutrophilic inflammation [3]. Other systemic markers demonstrating predictive value for mortality and exacerbation frequency include tumour necrosis factor (TNF-α) and the soluble receptor for advanced glycation end products (sRAGE) [30].

Neopterin, produced by activated macrophages in response to interferon-γ, reflects cell-mediated immunity against intracellular pathogens. The soluble triggering receptor expressed on myeloid cells-1 (sTREM-1) is elevated in sepsis and helps distinguish infectious inflammation from non-infectious inflammation. Other markers, including adrenomedullin (ADM), copeptin, endothelin-1 (ET-1), and natriuretic peptides (ANP and BNP), reflect vascular, metabolic, and cardiac stress responses and have roles in immune modulation and risk assessment [33].

Biomarkers of oxidative stress, such as hydrogen peroxide (H_2_O_2_) and 8-isoprostane, are increased in the exhaled breath concentrate (EBC), sputum, and systemic circulation of COPD patients. Oxidative stress is further increased during exacerbations. Oxidants are generated by both cigarette smoke and other inhaled particulates and are released from activated inflammatory cells such as macrophages and neutrophils [29].

In primary care settings where advanced cytokine profiling is economically or logistically nonviable, clinicians are now relying on cost-effective biomarkers derived from routine complete blood counts. The neutrophil-to-lymphocyte ratio (NLR) and the platelet-to-lymphocyte ratio (PLR) have been robustly validated as predictive indicators of high-risk COPD exacerbation. A comprehensive percentile-based risk stratification system utilizing the systemic immune-inflammation index (SII, which incorporates the NLR and PLR) successfully categorizes patients into distinct cohorts: a low-risk group (<290 points, yielding an annual exacerbation rate of 17%), an intermediate-risk group (290–768 points, 19.1% rate), and a high-risk group (>768 points, 23.4% rate). This scalable diagnostic strategy enables targeted management interventions in resource-constrained healthcare environments [34]. Surfactant proteins help distinguish true COPD pathology from other overlapping chronic lung diseases [3].

Beyond sterile inflammatory pathways, chronic airway dysbiosis characterized by a loss of microbial diversity and the proliferation of pathogenic genera such as *Haemophilus*, *Moraxella*, and *Pseudomonas* is now recognized as a primary catalyst for frequent exacerbations. This altered microbiome directly interacts with the airway epithelium to perpetuate a localized host-immune response, driving the persistent influx of neutrophils via sustained IL-8 release. Clinically, this microbial shift not only establishes the underlying chronicity of neutrophilic inflammation but also lowers the threshold for acute infectious exacerbations, further emphasizing the need for biomarkers that can effectively guide antimicrobial stewardship and target localized dysbiosis [35].

#### 2.2.3. Early Detection of COPD Symptoms

The underdiagnosis of COPD among patients is a major management challenge, with it staying undetected until they progress to a stage where lung damage is irreversible damage. The standard lower limit of normal (LLN) or fixed ratio is used in spirometry thresholds fails to detect early structural and functional changes in smokers’ airways. Researchers are validating extremely sensitive physiological techniques and molecular biomarkers to identify smokers who do not have any symptoms. The forced oscillation method (FOT), which gauges the resistance and reactance of the respiratory system during regular tidal breathing, is very useful for early detection of small airway disease.

In a cross-sectional study, smokers who had no symptoms and had perfectly normal spirometry lung function showed notably elevated FOT resistance parameters (specifically R5) when compared to healthy controls (*p* < 0.05). These physiological FOT parameters have a strong correlation with the underlying molecular mechanisms by which tissue is destroyed. Measurements of R5-R20, AX, and X5 demonstrated positive correlations with the levels of matrix metalloproteinase-9 (MMP-9) (*p* < 0.05) [6]. MMP-9, along with tissue inhibitors of metalloproteinases (TIMP-1 and TIMP-2), causes damage to the extracellular matrix, resulting in emphysema development [36]. Advanced gene expression profiling of lung tissue collected via laser capture microdissection revealed that genes related to repair and tissue damage are significantly increased in areas affected by emphysema, especially in small airways, creating a unique genetic pattern for the early stages of the disease [37]. Large studies, such as the KOL-Örestad study, regularly examine blood samples from smokers via advanced testing methods to develop tools that predict early lung function decline [38].

#### 2.2.4. Lung Cancer and COPD

Lung cancer surveillance is strongly linked to the need for early COPD detection [30]. Clinical data synthesized over the last two decades establish that the presence of COPD acts as a major, independent risk biomarker for lung cancer. Approximately 1% of patients diagnosed with COPD develop lung cancer annually, compared with only 0.2% of active or former smokers with normal lung function, representing a 5-fold increased risk [38]. Consequently, early biomarker-driven detection of COPD necessitates immediate and heightened radiological surveillance via CT scanning to diagnose lung cancer at its earliest, most curable stages [39].

Advanced imaging biomarkers, specifically quantitative computed tomography (QCT), which assesses airway remodelling and functional small airway disease, now serve as critical non-invasive markers bridging the diagnostic gap between chronic inflammation and oncogenesis [30]. Table 2 presents a structured overview of both established and emerging biomarkers used in the diagnosis, phenotyping, and management of COPD.

## 3. Biomarkers in Interstitial Lung Disease (ILD) & Idiopathic Pulmonary Fibrosis (IPF)

Interstitial lung diseases (ILDs) represent a heterogeneous collection of more than 200 distinct pulmonary disorders characterized by varying degrees of inflammation, progressive fibrosis, and unpredictable clinical outcomes. Idiopathic pulmonary fibrosis (IPF) is the prototype and most aggressive form of fibrosing ILD and is characterized by the continuous destruction of lung tissue, reduced pulmonary function, and a median survival rate of three to five years [40]. Similar progressive fibrosing phenotypes frequently complicate connective tissue diseases (CTD-ILDs), such as systemic sclerosis, rheumatoid arthritis, and idiopathic inflammatory myopathies. Despite their shared clinical presentations, these diseases exhibit different pathogenetic and immunologic microenvironments. The challenge in modern pulmonology is predicting an individual patient’s risk for rapid disease progression, acute exacerbation, or mortality to optimize the initiation of antifibrotic therapies and to stratify clinical trial designs [41].

Over the last ten years, many protein markers from cells, such as cancer antigen 19-9 (CA19-9), CA-125, osteopontin, and CXCL7, have been suggested through advanced testing methods to differentiate IPF from other ILDs [42]. However, without sufficient large-scale validation, these novel markers lack the evidence required for inclusion in international guidelines [43].

Comprehensive biomarker analyses of the ISABELA 1 and 2 clinical trials recently bridged this critical validation gap. This dataset comprises longitudinal plasma samples from 1280 IPF patients and represents the largest and most definitive IPF biomarker cohort evaluated to date. Using advanced statistical methods to study 17 disease-related biomarkers and the MUC5B genotype, researchers identified matrix metalloproteinase-7 (MMP-7) and C-C motif chemokine ligand 18 (CCL18) as prognostic biomarkers for IPF [44].

Matrix metalloproteinase-7 (MMP-7) is a proteolytic enzyme involved in extracellular matrix (ECM) remodelling and is predominantly expressed by hyperplastic alveolar epithelial cells situated within active fibroblast foci [42]. In the ISABELA cohort, baseline MMP-7 levels were significantly elevated in patients who later developed a rapid, ≥10% annual decline in forced vital capacity (FVC) compared with those with slower progression (median 5.5 µg/L vs. 4.2 µg/L; *p* < 0.005). Patients with baseline MMP-7 levels greater than 5.2 μg/L were identified as a high-risk group, with a significantly higher likelihood of all-cause mortality (*p* < 0.0001), (*p* = 0.0001), comprehensive disease progression (*p* < 0.01) and respiratory-related admissions. This high-risk marker remained significant even for patients who were being treated with standard antifibrotic medications such as nintedanib or pirfenidone, indicating that MMP-7 is a separate indicator of severe disease [44].

C-C motif chemokine ligand 18 (CCL18) is a chemokine produced primarily by activated macrophages, reflecting severe, ongoing profibrotic immunological signalling [42]. The ISABELA trials revealed that baseline elevations in CCL18 (≥75.2 µg/L) were associated with significantly increased risks of mortality (*p* < 0.0001) and respiratory hospitalizations. Machine learning models revealed that longitudinal fold changes in CCL18 levels measured at week 26 served as the strongest soluble predictor of short-term disease progression. Furthermore, acute, life-threatening exacerbations of IPF are best predicted by a combination of rapid fold changes in CCL18, CCL-2, and CXCL13. Multivariable machine learning models incorporating these longitudinal chemokine changes alongside established baseline clinical parameters specifically the diffusing capacity of the lungs for carbon monoxide (DLCO%) demonstrated significant prognostic accuracy for disease progression (c-index: 0.67) [44].

The clinical integration of these two markers helps in risk stratification. IPF patients presenting with dual biomarker elevation, high baseline MMP-7 (≥5.2 µg/L), and high CCL18 (≥75.2 µg/L) levels face a high risk of short-term mortality (*p* < 0.00001) [44]. While MMP-7 is an excellent prognostic indicator, researchers advise caution that its expression is not exclusively unique to IPF. However, beyond protein mediators, leukocyte telomere length has emerged as a thoroughly validated molecular biomarker. Shorter telomere lengths are independently associated with worse transplant-free survival and an accelerated clinical decline in patients with IPF, establishing it as a highly reliable tool for individualized risk stratification and prognostic assessment [45]. Hence, the future of ILD diagnosis will rely on integrated multimarker panels and quantitative CT imaging utilizing deep learning classification systems to differentiate typical interstitial pneumonia (UIP) patterns and predict outcomes independently of visual assessments [43].

In a major recent regulatory milestone, the PROLIFIC risk score developed by the Prognostic Lung Fibrosis Consortium to predict 1-year disease progression became the first IPF biomarker tool to be officially accepted by the FDA Center for Drug Evaluation and Research (CDER) Biomarker Qualification Program, accelerating the validation of prognostic markers such as SP-D, CXCL13, TNC, and MMP-7 for clinical trial enrichment. Furthermore, advanced gel-free quantitative proteomics of bronchoalveolar lavage fluid (BALF) has revealed additional upregulated mediators in IPF, including osteopontin, CXCL7, and the profibrotic cytokine CCL24 [42].

Among the circulating proteins evaluated in interstitial lung disease (ILD), Krebs von den Lungen-6 (KL-6), an MUC1 mucin produced by regenerating type II pneumocytes, has been established as a biomarker of disease activity, particularly in Japan, where it is routinely used in clinical practice [46]. Serum KL-6 levels correlate with disease severity in patients with fibrotic ILDs, especially those associated with underlying systemic autoimmune conditions, and elevated levels have been shown to predict acute exacerbations [40].

The findings of a systematic review indicated that serum YKL-40 levels were elevated in patients with idiopathic pulmonary fibrosis, connective tissue disease-associated ILD, sarcoidosis, cryptogenic organizing pneumonia, asbestosis-related ILD, and idiopathic nonspecific interstitial pneumonia compared with controls, whereas no increase was observed in patients with pulmonary alveolar proteinosis. Additionally, serum YKL-40 levels vary across different ILD subtypes. Furthermore, YKL-40 concentrations in bronchoalveolar lavage fluid were significantly higher in patients with idiopathic pulmonary fibrosis than in controls [47].

### Autoimmune and Connective Tissue ILDs

For patients presenting with ILDs characterized by autoimmune features (IPAF) or distinct connective tissue disease aetiologies (CTD-ILD), the biomarker focus shifts toward markers of alveolar epithelial damage and the innate immune response, primarily Krebs von den Lungen-6 (KL-6) and surfactant protein D (SP-D). KL-6 is a high-molecular-weight mucin-like glycoprotein highly expressed on proliferating type II pneumocytes, whereas SP-D is a large hydrophilic collection involved in pulmonary host defence [40,46].

In well-characterized CTD-ILD cohorts, the serum levels of both KL-6 and SP-D are significantly positively correlated with bronchoalveolar lavage (BAL) cellularity counts (KL-6: r = 0.58, *p* = 0.04; SP-D: r = 0.63, *p* = 0.02) and parallel the severity of fibrotic changes detected via high-resolution computed tomography (HRCT). KL-6 expression was negatively correlated with the time elapsed since initial symptom onset (r = −0.51, *p* = 0.02), suggesting increased activity in the early acute inflammatory phase. There is an inverse correlation between KL-6 and SP-D levels and overall exercise capacity and baseline pulmonary function tests in patients with IPAF [48].

Despite these clear associations with disease severity and lung impairment, the clinical applicability of SP-D and KL-6 is still limited due to enormous variation in sensitivity and specificity across various autoimmune subsets. These markers cannot currently be used as highly reliable, standalone predictors of short-term fibrotic progression or immediate treatment response. As antifibrotics (nintedanib) and targeted biologics (rituximab, tocilizumab) have become part of the therapeutic landscape for immunologically active variants of CTD-ILDs, integrative biomarker panels that combine KL-6/SP-D with particular autoantibodies (e.g., anti-MDA5) and chemokines (CXCL4) are urgently needed to improve patient stratification [41]. Table 3 gives a structured summary of established and newer biomarkers used in ILD diagnosis, phenotyping and management.

## 4. Biomarkers in Lung Cancer

Lung cancer remains the leading cause of cancer-related mortality and is the second most common cancer diagnosed worldwide. An estimated 2.2 million new cases and 1.8 million deaths resulted in significant social and financial expenses. Based on histological classification, lung cancer can be broadly classified as either small-cell lung cancer or non-small cell lung cancer (NSCLC). The most prevalent subtypes of non-small cell lung cancer (NSCLC) which accounts for approximately 85% of all cases, are squamous cell carcinoma and adenocarcinoma. Despite significant improvements in surgical techniques, radiotherapy, chemotherapy, targeted therapies and immunotherapy the overall 5-year survival rate for patients with lung cancer remains low at approximately 19% [49].

High-risk populations have shown success with low-dose computed tomography (LDCT) screening, indicating a 20–24% decrease in mortality (as evidenced by the landmark US-based National Lung Screening Trial (NLST) and the Dutch–Belgian lung cancer screening trial (NELSON trials) but systemic LDCT implementation has drawbacks of its own [49]. Fewer than 18% to 20% of eligible individuals in the United States currently undergo screening, which is due to reduced accessibility, persistent disease stigma, and rigid United States Preventive Services Task Force (USPSTF) eligibility criteria (focusing heavily on individuals aged 50–80 years with ≥20 pack-year histories), which by default excludes high-risk nonsmokers [10]. Furthermore, LDCT has high false-positive rates; the majority of detected pulmonary nodules are benign, yet their identification triggers intense anxiety and mandates expensive, invasive diagnostic tests. Invasive procedures, such as CT-guided biopsies, carry substantial risks (e.g., a 30% pneumothorax rate), and an analysis of health economics indicates that 43.1% of the total cost of lung cancer laboratory tests is directly attributed to invasive biopsies [49].

To overcome these limitations, the field has rapidly shifted toward minimally invasive liquid biopsies. These biopsies analyse peripheral blood, urine, or cerebrospinal fluid to detect tumour-derived cells shed into the circulation, including circulating tumour cells (CTCs), circulating tumour DNA (ctDNA), extracellular vesicles (EVs), and microRNAs (miRNAs) [11]. Liquid biopsies overcome the challenges of tissue biopsies, providing a dynamic, real-time snapshot of the global tumour burden [50]. In advanced non-small cell lung cancer (NSCLC), ctDNA analysis is now a critical, mandated biomarker test in clinical practice. In order to find actionable parameters, the 2026 American Society of Clinical Oncology (ASCO) guidelines stress the significance of thorough molecular profiling using next-generation sequencing (NGS), frequently combining tissue and ctDNA (e.g., the g. EGFR mutations’ MET amplification, ROS1 rearrangements, HER2 exon 20 mutations) and direct individualized therapy [51]. Additionally, the precise identification of CNS metastatic profiles and drug resistance mechanisms is made possible by the analysis of ctDNA extracted from cerebrospinal fluid (CSF), which improves diagnostic sensitivity for leptomeningeal disease. Also, the measurement of circulating tumour cells (CTCs) has prognostic value, in particular a poor prognosis is strongly correlated with ≥5 CTCs per 7.5 mL of blood [52].

The clinical utility of ctDNA profiling in early-stage disease (Stages I–IIIA) remains constrained by low diagnostic sensitivity (~0.27) secondary to minimal tumor shedding and rapid systemic clearance [53]. To address this diagnostic gap, stable circulating microRNAs (miRNAs) are emerging as highly specific biomarkers for early malignancy [9]. Notably, a recently validated 6-miRNA serum panel outperformed traditional risk models; when deployed as an adjunct to LDCT for indeterminate nodules, it enhances clinical risk stratification, mitigates false-positive rates, and curtails unnecessary invasive biopsies [54].

In addition to genetic mutations and circulating microRNAs, epigenetic biomarkers most notably DNA methylation profiling are increasingly recognized as critical tools for early detection and disease classification. Aberrant DNA methylation, such as the hypermethylation of tumor-suppressor gene promoters, is a foundational event in lung tumorigenesis that often precedes detectable genetic mutations. Because these methylation signatures are highly tissue-specific and are faithfully preserved in circulating tumor DNA (ctDNA), they provide a robust liquid biopsy medium for distinguishing malignant from benign pulmonary nodules and classifying disease subtypes [55]. High-throughput targeted DNA methylation sequencing of ctDNA has demonstrated exceptional sensitivity and specificity in detecting early-stage lung cancer, establishing epigenetic profiling as a highly promising and complementary approach to low-dose CT screening [56].

In line with liquid biopsy advancements, professional radiologic societies have rigorously updated their structured reporting frameworks (such as Lung-RADS v2022) and surveillance algorithms to manage the growing volume of incidentally discovered and screening-detected pulmonary nodules [49]. The 2025 guidelines issued by the National Comprehensive Cancer Network (NCCN) and the European Society of Thoracic Imaging (ESTI) establish highly precise, morphometrically driven follow-up protocols. ESTI guidelines rely heavily on precise volumetric measurements (mm^3^) and volume doubling times (VDTs). A new nodule demonstrating >15% volume growth or >1.5 mm linear diameter growth within 3 months demands immediate referral for clinical workup. Similarly, prevalent nodules exhibit rapid exponential growth (VDT < 250 days after 3 months, <400 days after 6 months, or <500 days after 12 months) or an average diameter increase >1.5 mm/year mandate immediate intervention. Conversely, the NCCN 2025 guidelines dictate immediate referral for prevalent nodules ≥15 mm in size presenting with high clinical suspicion or for growing nodules ≥8 mm in size [57]. For smaller, unsuspicious lesions (<6 mm NCCN or <100 mm^3^ ESTI), conservative 12-month routine CT surveillance remains the standard of care [57].

The landscape of lung cancer therapy has been revolutionized by immune checkpoint inhibitors (ICIs), with PD-L1 expression serving as the primary validated biomarker for treatment selection. Clinical protocols now categorize patients based on Tumor Proportion Score (TPS) thresholds, with a TPS ≥50% generally identifying patients who may benefit from ICI monotherapy. However, PD-L1 remains an inaccurate predictor, therefore, contemporary diagnostic frameworks are increasingly integrating Tumor Mutation Burden (TMB) as a complementary biomarker to improve the precision of immunotherapy patient selection [58].

Table 4 presents a structured overview of both established and emerging biomarkers used in the diagnosis, phenotyping, and management of lung cancer as per the latest National Comprehensive Cancer Network (NCCN) Clinical Practice Guidelines in Oncology [58,59].

## 5. Biomarkers in Bronchiectasis and Cystic Fibrosis

The EMBARC-BRIDGE study characterized the eosinophilic endotype of noncystic fibrosis bronchiectasis, which is clinically defined by a blood eosinophil count of ≥300 cells/µL. Advanced liquid chromatography tandem mass spectrometry (LC–MS/MS) of sputum from these patients revealed increased concentrations of eosinophil proteins, including galectin-10, eosinophil cationic protein (ECP), and eosinophil-derived neurotoxin (EDN). These airway biomarkers correlate with Aspergillus sensitization, Pseudomonas aeruginosa infection and radiological severity, suggesting that measuring airway inflammation directly provides more information than basic measurements of peripheral blood. This has been observed across various demographic cohorts with different aetiologies. In contrast, serum tumour necrosis factor-alpha (TNF-α) has become a significant biomarker of severity, linked to microbial colonization and exacerbations. In patients with cystic fibrosis (CF), the focus has shifted to long-term comorbidities and novel blood biomarkers as a result of CFTR modulators therapy. Additionally urinary biomarkers alongside urinary extracellular vesicles are now being utilized to detect CF-related kidney disease (CFKD) in its early stages [60].

## 6. Biomarkers in Pulmonary Vascular Diseases

### 6.1. Acute Pulmonary Embolism (PE) and Pulmonary Arterial Hypertension (PAH)

The American Heart Association (AHA) and the American College of Cardiology (ACC), in their joint clinical practice guidelines from 2026, have entirely revised the acute risk stratification of acute pulmonary embolism. The guidelines introduce a five-tier framework (Clinical Categories A through E) that relies on integrated biomarker assessments. D-dimer remains a validated biomarker for excluding thromboembolism in low-risk patients and predicting the severity of respiratory infections. Cardiac biomarkers, specifically troponin and B-type natriuretic peptide (BNP), are utilized to identify patients at high risk of short-term complications and mortality, whereas serum lactate is mandated to detect subclinical end–organ hypoperfusion in normotensive patients, guiding escalation to systemic or catheter-directed thrombolysis [61].

### 6.2. Pulmonary Arterial Hypertension (PAH)

In chronic vascular disease, particularly pulmonary arterial hypertension (PAH), BNP and NT-proBNP remain the only fully validated prognostic markers. However, transpulmonary exosomal microRNAs (such as miR-21 and miR-8078) have emerged as novel indicators reflecting the pathogenic microenvironment of the pulmonary circulation, highlighting new therapeutic targets. Additional investigated markers include red cell distribution width (RDW) and Cystatin C [62].

## 7. Biomarkers in Pulmonary Infections & Acute Respiratory Distress Syndrome (ARDS)

The heterogeneity of ARDS has driven researchers to identify distinct biomarker-based sub-phenotypes to guide therapy. Patients stratified into a “hyperinflammatory” sub-phenotype via inflammatory biomarker panels face a significantly greater risk of mortality (RR 2.50) than their hypo inflammatory counterparts do. Elevated levels of the soluble receptor for advanced glycation end products (sRAGE) above 16,500 pg./mL serve as strong predictors of 28-day mortality and alveolar epithelial injury [63].

Furthermore, deep metabolomic profiling revealed that ARDS pathogenesis is heavily characterized by metabolic reprogramming, specifically indicated by elevated phenylalanine and lactate levels, alongside depletion of sphingosine 1-phosphate and citrulline [64].

In infectious respiratory contexts, particularly Community-Acquired Pneumonia (CAP), diagnostic delays are mitigated by host-response transcriptomics. Novel gene expression signatures comprising 3 to 10 specific transcripts can now accurately differentiate atypical *Mycoplasma pneumoniae* infections from other bacterial and viral pneumonias with high accuracy (AUC ranging from 0.84 to 0.95), drastically reducing the empirical overprescription of beta-lactam antibiotics [65]. In severe CAP, tracking systemic inflammatory markers such as IL-6, IL-8, IFN-γ, and G-CSF provides a highly effective method for monitoring treatment response and forecasting hospitalization risk [66]. For CAP patients, regular examinations of procalcitonin can help adjust the course of antibiotics, that is, the time to stop antibiotics. Ito et al. demonstrated that procalcitonin can guide the duration of antibiotic use in CAP patients from 12.6 days to 8.6 days [67].

While peripheral blood and induced sputum represent traditional pillars of biomarker sampling, the respiratory tract offers a uniquely direct, continuous, and entirely non-invasive diagnostic medium, i.e., human breath. The rapidly expanding field of “breath omics” focuses on the intricate biochemical matrix expelled during normal tidal breathing, which is broadly categorized into gas-phase volatile organic compounds (VOCs) and the aerosolized liquid phase known as exhaled breath condensate (EBC) [68].

EBC is generated by cooling and condensing water vapour and aerosolized microscopic droplets that originate directly from the airway lining fluid (ALF) of the lower respiratory tract. This entirely non-invasive procedure exerts zero influence on underlying airway function or inflammation but captures an incredibly rich, organ-specific biofluid. The EBC matrix contains a vast array of non-volatile components that reflect local respiratory biology. These include inflammatory mediators (e.g., eicosanoids, leukotrienes, and isoprostanes), reactive oxygen species (hydrogen peroxide [H_2_O_2_]), simple ions (ammonia and nitrogen oxides), complex epithelial-derived proteins (cytokines, ezrin, and periostin), and cell-free nucleic acids (cfNAs), including genomic DNA, mitochondrial DNA, messenger RNA, and microbial genetic material [68].

Advanced molecular profiling of EBC via high-throughput metabolomics and proteomics, specifically nuclear magnetic resonance (NMR) and mass spectrometry (MS), has proven highly capable of discriminating among complex disease phenotypes. NMR offers excellent reproducibility with minimal sample preparation, identifying distinct metabolic types and tracking longitudinal treatment responses. MS-based approaches provide excellent sensitivity, enabling granular profiling of amino acids derivatives and eicosanoids derived from lipids in groups of people with asthma. A lower pH linked to active disease and altered eicosanoid concentrations were identified by EBC analysis. Elevated proinflammatory periostin levels, decreased ezrin expression and airway inflammation demonstrate EBCs significant potential for non-invasive endotype discrimination and in monitoring treatment effectiveness [21].

Apart from chronic inflammatory diseases, breath omics has shown promise as a diagnostic tool for infections and cancerous diseases [69]. In the VICTORY study, advanced portable sensor technologies (e.g., Inflammacheck^®^ device manufactured by Exhalation Technology Ltd, headquartered in Cambridge, United Kingdom) were used. Seven different EBC variables were assessed by researchers including peak CO_2_ percentages, H_2_O_2_ levels and exhalation flow rates. The study confirmed that elevated exhaled H_2_O_2_ levels increased the predictive likelihood of diagnosing pneumonia (by 25.7-fold) and lung cancer (by 3.6-fold), establishing multiparameter EBC signatures as robust diagnostic classifiers [70]. The integration of these molecular EBC indices with established clinical biomarkers of T2 inflammation (FeNO, peripheral eosinophils, IgE) promises a multimodal approach that significantly improves the predictive accuracy of reversible airway obstruction and bronchodilator reversibility (BDR) [71].

Despite the immense diagnostic potential of breath omics, significant translational barriers currently limit its integration into routine clinical workflows. A primary obstacle is the absence of universally standardized collection protocols, which leads to substantial variability in sample yields and complicates cross-study comparisons. Furthermore, the highly sensitive biochemical matrix of exhaled breath condensate (EBC) is remarkably susceptible to confounding by ambient air contaminants, environmental humidity, and variations in patient exhalation flow rates. Until these environmental and methodological inconsistencies are addressed through rigorous, standardized point-of-care calibration, breathomics will remain largely investigational rather than a reliable diagnostic fixture in daily patient management [72].

## 8. Biomarkers in Neonatal and Pediatric Lung Diseases

Biomarkers in neonatal lung disease serve as crucial biological indicators for the early diagnosis, monitoring, and prognosis of conditions such as respiratory distress syndrome (RDS) and bronchopulmonary dysplasia (BPD) [73]. Among the most commonly studied biomarkers in neonates are inflammatory and immune markers, which are tested in blood or tracheal aspirates to assess the severity of pulmonary inflammation. Elevated levels of interleukin-6 (IL-6) and IL-8 are closely associated with lung injury and indicate a high risk for developing BPD [74]. Additionally, tumour necrosis factor-alpha (TNF-α) interrupts normal alveolar and vascular development through inflammatory pathways, whereas TGF-β acts as a negative regulator of alveolar development, affecting late-stage lung development and promoting fibrosis [75].

Angiogenic growth factors are critical biomarkers, as disrupted vessel growth frequently leads to BPD and BPD-associated pulmonary hypertension. Vascular endothelial growth factor (VEGF) and endothelin 1 play vital roles in pulmonary angiogenesis, and reduced or abnormally altered levels are related to aberrant vascular development [75]. To evaluate the risk of pulmonary hypertension and cardiac functioning in infants with severe BPD, clinicians also rely on B-type natriuretic peptides, specifically BNP and NT-pro-BNP [76].

As preterm lungs are highly vulnerable to free radical damage, oxidative stress markers are widely utilized to assess tissue injury. Lipid peroxidation is measured via 8-iso-prostaglandin F_2_α and malondialdehyde (MDA) levels, both of which indicate damage to cell membranes. For assessing DNA damage, 8-hydroxy-2-deoxy guanosine (8-OHdG) serves as a reliable marker. Moreover, advanced oxidation protein products (AOPPs) and protein carbonyls are examples of markers of protein oxidation [73]. AOPPs are used to detect more extensive tissue damage associated with oxidative stress [74].

Important diagnostic information is also provided by surfactants and specific markers. Lung maturity has historically been evaluated using the lecithin/sphingomyelin (L/S) ratio, with a ratio of less than 2.2 being a crucial clinical marker for RDS [77]. At present microRNAs like miR-92 and miR-122 as well as proteins like ANGPTL4 are under investigation for the prediction and treatment of lung diseases in newborns [78]. Genetic screening, as well as mutations in the ABCA3 gene, which controls the pulmonary surfactants metabolism, helps identify at-risk individuals in neonates and provides direct guidance for clinical interventions [79].

In the context of evaluating airway epithelial damage, Club Cell Secretory Protein (CC16) has emerged as a critical clinical indicator of neonatal lung injury. Predominantly expressed in the airways, circulating CC16 acts as a surrogate marker for epithelial barrier leakage into the periphery. Clinically, reduced circulating levels of CC16 reflect the depletion of protective club cells and are strongly associated with longitudinal lung function deficits. This establishes CC16 as a valuable prognostic biomarker for identifying premature infants at an elevated risk of developing bronchopulmonary dysplasia (BPD) and long-term pulmonary complications [80].

Lung Ultrasound Scores (LUS) and omics profiling are two non-invasive diagnostic techniques that significantly alters how clinicians anticipate and treat lung disorders in newborns. Neonates are particularly susceptible to ionizing radiation from repeated X-rays. A crucial and secure substitute for CT scans is point-of-care lung ultrasound (POCUS) in the NICU [81]. In infants with neonatal respiratory distress syndrome (NRDS), an elevated LUS calculated within the first hours of life can predict the need for surfactant replacement therapy before oxygen levels decrease [82]. Furthermore, LUS provides guidance for ventilator weaning; semiquantitative tracking allows neonatologists to monitor lung reaeration, where a low, stable score (typically ≤ 1) indicates that an infant’s lungs have recovered enough to be safely weaned from continuous positive airway pressure (CPAP) or mechanical ventilation. Serial LUS tracking also facilitates early screening for BPD caused by oxygen toxicity and mechanical trauma. By identifying structural lung changes early, clinicians can alter ventilation settings before permanent injury occurs [81].

Concurrently, the NICU’s shift toward non-invasive, gentle ventilation has made traditional biological samples, such as tracheal aspirates, less available. In response, researchers are depending on advanced omics techniques involving completely non-invasive biofluids, including urine, oral secretions, and umbilical cord blood. Through high-throughput mass spectrometry, and proteomics oral secretions and urine from premature infants are analysed to identify altered protein patterns. Specifically, proteins associated with neutrophil degranulation and immune dysregulation, such as SERPINC1 and BPI, serve as early warnings for BPD development [83]. Moreover, peripheral blood transcriptomics can be used to predict gene expression signatures. By studying messenger RNA (mRNA) in microvolume in neonatal blood, machine learning algorithms can map early genetic profiles. This transcriptomic profiling stratifies newborn risk categories, identifying which premature infants possess high genetic susceptibility to severe respiratory distress or chronic lung outcomes [84].

## 9. Artificial Intelligence and Multiomics in Respiratory Disease Biomarker Research

The contemporary era of respiratory biomarker research is defined not only by the discovery of novel biological entities but also by the multidimensionality of the data generated across multiomics fields (genomics, epigenomics, transcriptomics, proteomics, metabolomics, and microbiomics) [7]. As the human analytical capacity is insufficient to interpret these vast, untargeted datasets, artificial intelligence (AI) has emerged as an important computational bridge that transforms complex, discrete datasets into clinically actionable insights and robust predictive models [10].

AI is being used in a variety of specialized fields for respiratory biomarker validation, with subdomains each suited to particular modalities of data [85].

**Machine Learning (ML):** ML algorithms (e.g., random forest, logistic regression, support vector machines and gradient boosting/XGBoost) are well suited for predictive and nonlinear learning as well as for identifying connections between electronic health records (EHR) and structured biomarker datasets. For instance, ML platforms that incorporate standard blood biomarkers (ferritin, haemoglobin, LDH and CRP) alongside vital signs have proven to be highly accurate in identifying respiratory conditions and disease progression mapping complex correlations that align with known inflammatory and metabolic effects [86]. Additionally, ML plays a key role in predicting acute asthma and in deriving interactive logistic-based risk scores for oncology patients undergoing targeted treatment [85]. A prospective study of 395 patients treated with bevacizumab analysed pretherapeutic clinical and laboratory data via multiple machine learning models. The random forest model (80/20 split) performed best (AUC-ROC = 0.75) and was used to develop a well-calibrated logistic risk score (AUC-ROC = 0.72). The key predictors included age ≥65 years, anaemia, elevated urea, leucocytosis, tumour differentiation, and stage. The final outcome is a simple offline tool that estimates individual complication risk and stratifies patients into risk categories, supporting personalized clinical decision-making [87].

**Deep Learning (DL):** DL, which uses complex multilayer neural networks (e.g., Convolutional Neural Networks, Transformers), is utilized for high-dimensional, unstructured inputs such as CT imaging and radiomics. DL architectures enable the automated, precise quantification of emphysema severity in COPD patients and the objective subtyping of fibrotic patterns in interstitial lung diseases [85].

**Natural Language Processing (NLP) & Large Language Models (LLMs):** These technologies automate the extraction of structured phenotypic variables and biomarker data within free-text clinical notes, accelerating the retrospective validation of asthma prediction indices and supporting real-time clinical documentation [85].

Furthermore, the integration of advanced concepts such as “digital twins”, which are virtual, highly detailed, data-driven replicas of patient physiological states, allows researchers to simulate high-dimensional multimodal data. In a large-scale modelling study involving lung and breast cancer datasets, digital twin simulations were adopted via Parzen–Rosenblatt constrained isometric mapping combined with radiomics (CT/PET) with genomics. This approach successfully assessed the adaptability of overall survival (OS) predictions and diagnostics under simulated biomarker fluctuation scenarios (±10%), advancing the robustness of data-driven decision-making in precision oncology [69].

## 10. Implementation Challenges and Future Directions

Beyond single-biomarker utility, the clinical management of chronic lung disease is shifting toward multidimensional scoring systems that integrate molecular, clinical, and radiological variables. Established benchmarks such as the Gender, Age, Physiology (GAP) Index remain the gold standard for predicting mortality in ILD, while the Body mass index, Airflow obstruction, Dyspnea, and Exercise capacity (BODE) Index continues to guide COPD management. Emerging prognostic models now augment these clinical scores with longitudinal chemokine measurements (e.g., CCL18) or epithelial injury markers (e.g., CC16), creating a more robust framework for risk-stratifying patients and tailoring therapeutic interventions [88,89,90,91].

Despite achieving unprecedented predictive performance in silico, the successful, real-world deployment of AI-driven biomarker systems into point-of-care clinical workflows remains a challenge with translational barriers [10]. A comprehensive 2025 scoping review that evaluated clinically validated AI-driven decision support tools in acute respiratory failure settings highlighted several deficiencies. While 67% of the eligible studies successfully predicted outcomes such as weaning from mechanical ventilation, the studies met a median of only 3.5 out of the 17 criteria mandated by the DECIDE-AI evaluation framework. Not a single study reported on AI-related errors, algorithmic malfunctions or vital considerations regarding ethnic bias and algorithmic fairness in the training set. Only a small percentage of studies have evaluated user adherence or human–computer agreement metrics, highlighting the enormous disparity. between clinical usefulness and computational design [92].

Decentralized architectures are being used in the field to overcome key obstacles, such as centralized implementation infrastructures, through federated learning frameworks (e.g., the ACR. RISE database). These systems enable the training of AI models across various global populations, improving generalizability without violating laws protecting patient privacy. In future multiomic biomarker panel validation will be subject to stringent compliance with established reporting standards such as TRIPOD+AI PROBAST+AI and CONSORT-AI guaranteeing that predictive algorithms are fair transparent and able to build the clinician trust needed to transform respiratory diagnostics [10].

A critical component of bridging the gap between computational prediction and clinical action is the implementation of Explainable AI (XAI). Unlike conventional ‘black-box’ deep learning models that generate outputs without justifying their decision-making process, XAI frameworks provide the transparency necessary to build clinician trust and ensure ethical accountability. In the context of respiratory biomarker research, XAI methods including feature attribution maps and saliency visualization allow clinicians to visualize the specific physiological or molecular data points driving an algorithmic prediction (e.g., highlighting specific radiographic regions or biomarker concentrations). This interpretability is vital for identifying potential algorithmic bias and ensuring that clinical decisions remain grounded in observable patient physiology. As emphasized in recent clinical standards, XAI is not merely a technical preference but a regulatory and ethical requirement for the safe, transparent integration of AI-driven diagnostic tools into frontline respiratory care [93].

## 11. Conclusions

During 2025–2026, precision diagnostics played a significant role in respiratory medicine, with targeted treatments based on approved biomarkers included in key clinical recommendations. The treatment of asthma and COPD now concentrates on particular inflammatory and molecular endotypes as opposed to methods based on symptoms. In severe conditions like pulmonary fibrosis and lung cancer, biomarkers enable better diagnosis and early risk assessment with innovations like multiomics and liquid biopsy that improve detection accuracy. Translating complex biological data into useful customized clinical judgment requires AI-driven analysis and standardized methodologies.

## Figures and Tables

**Table 1:** Established and Emerging Asthma Biomarkers. * Blood Eosinophil cutoff ≥ 300 cells/μL (high risk). ** FeNO cutoff > 50 ppb (high T2 inflammation). Other biomarker cutoffs are assay-dependent.

Biomarker	Primary Biological Source	Pathophysiological Association	Clinical Utility
Blood Eosinophils *	Peripheral Blood	Type 2 inflammation; exacerbated risk	Guides anti-IL-5/IL-4R therapies. Standard Care (GINA 2026) [15].
Sputum Eosinophils	Induced Sputum	Type 2 (Eosinophilic)	Gold standard for measuring airway eosinophilia; predicts exacerbation risk [15].
Total Serum IgE	Blood (Serum)	Type 2 (Allergic)	Identifies allergic phenotype; determines eligibility and dosing for anti-IgE therapy [15].
Specific IgE	Blood/Skin	Type 2 (Allergic)	Identifies specific environmental aeroallergen triggers driving the allergic response [15].
Fractional exhaled nitric oxide (FeNO) **	Exhaled Breath	Type 2 inflammation, IL-13-driven epithelial inflammation	Phenotype allocation (20 ppb). Predicts steroid responsiveness and guides anti-IL-4/13 biologic therapy [15].
Sputum IL-13	Induced Sputum	Localized T2 cytokine signalling	Superior marker for asthma control; predicts omalizumab response. Investigational surrogate marker [17].
Sputum IL-33	Induced Sputum	Type 2 inflammation, Neuro-inflammatory interactions	Linked to mixed granulocytic asthma and capsaicin sensitivity. Investigational surrogate marker [17].
Galectin-10	Serum, Sputum, EVs	Type 2 inflammation, Charcot-Leyden crystal formation	Correlates with fixed airflow limitation and SAD. EV-Galectin-10 superior to blood eosinophils. Last-stage validation [19].
Periostin	Serum, EBC	Type 2 inflammation, IL-13-driven airway inflammation, and tissue remodelling	Indicates active airway remodelling. Investigational surrogate marker [14].
Urinary Leukotriene E4 (uLTE4)	Urine	Type 2 inflammation, Aspirin-Exacerbated Respiratory Disease (AERD)	Highly elevated in Aspirin-Exacerbated Respiratory Disease; marks leukotriene overproduction [22].
Eosinophil Cationic Protein (ECP)	Blood/Sputum	Type 2 (Eosinophilic)	Reflects active eosinophil degranulation; correlates strongly with asthma severity [20].
Sputum Neutrophils	Induced Sputum	Non-Type 2 (Neutrophilic)	Indicates non-T2 inflammation; often associated with severe corticosteroid resistance [14].
YKL-40	Blood/Sputum	Non-Type 2	Emerging marker associated with airway remodelling, severity, and treatment resistance [25].
Epithelial Alarmins (e.g., TSLP, IL-33, IL-25)	Sputum, Serum, BAL, Exhaled breath	Upstream mediators of airway inflammation; released upon epithelial injury to orchestrate both T2 and non-T2 cascades.	Emerging indicators of disease severity and airway hyperresponsiveness; target for broad-acting biologic therapies (e.g., Tezepelumab) across various endotypes [27,28].

**Table 2:** Cellular and Molecular Biomarkers in COPD. * Blood Eosinophils cutoff ≥ 300 cells/μL (high risk). Other biomarker cutoffs are assay-dependent.

Biomarker	Primary Biological Source	Physiological/Pathological Association	Clinical Utility & Validation Status
Blood Eosinophils *	Peripheral Blood	Systemic eosinophilic inflammation.	Predicts exacerbation risk; widely used to guide the initiation and withdrawal of inhaled corticosteroid (ICS) therapy. GOLD 2026 standard dictates ICS usage and biologic initiation (300 cells/µL) [6].
C-Reactive Protein (CRP)	Liver (acute-phase proteins), Blood	Systemic acute-phase inflammatory response.	Routinely utilized to evaluate the severity of an exacerbation and overall patient prognosis [3].
Procalcitonin (PCT)	Thyroid C-cells and neuroendocrine cells, Peripheral Blood	Systemic response specifically to bacterial infection.	Assists in differentiating bacterial exacerbations from viral ones; highly useful for guiding judicious antibiotic stewardship [30]
Fibrinogen	Peripheral Blood	Systemic acute-phase protein elevation and coagulation cascade activation.	Serves as a validated, FDA-qualified biomarker for assessing disease severity and predicting exacerbation risk in clinical trials [3].
Inflammatory Ratios (NLR, PLR, SII)	Peripheral Blood	Systemic immune-inflammatory imbalance (e.g., elevated neutrophils vs. reduced lymphocytes).	Readily accessible composite indices used to identify patients at a high risk for frequent exacerbations [34].
Neopterin	Peripheral Blood	Produced by activated macrophages in response to interferon-γ; reflects cell-mediated immunity against intracellular pathogens.	Helps indicate viral or atypical intracellular infections during acute exacerbation episodes [33].
Soluble Triggering Receptor Expressed on Myeloid Cells-1 (sTREM-1)	Peripheral Blood/Sputum	Soluble receptor upregulated and shed by myeloid cells during severe acute inflammatory responses.	Elevated in sepsis; crucially helps distinguish infectious from non-infectious inflammation [33].
Stress Markers (ADM, Copeptin, ET-1, ANP, BNP)	Peripheral Blood	Reflects complex vascular, metabolic, and cardiac stress responses secondary to respiratory insufficiency.	Holds documented clinical roles in immune modulation, cardiovascular risk profiling, and advanced clinical risk assessment [33].
Sputum Eosinophils	Induced Sputum	Localized, eosinophil-driven airway inflammation.	Correlates directly with accelerated lung function decline, more severe emphysema, and a heightened risk for acute exacerbations [33].
Cytokines (IL-1α, IL-1β, TNF-α, IL-17A)	Induced Sputum	Pro-inflammatory cascades, often heavily upregulated during bacterial infection.	Facilitates the specific phenotyping of exacerbations to determine underlying bacterial triggers [33].
IL-6 & IL-8	Sputum/BAL	Potent neutrophil chemotaxis (IL-8) and localized chronic tissue inflammation.	Investigational markers used to monitor localized inflammation and structural damage progression [3].
Proteases (MMPs & Neutrophil Elastase)	Induced Sputum	Protease–antiprotease imbalance directly driving structural alveolar wall destruction.	Investigational biomarkers for assessing the progression and severity of emphysematous destruction [6].
Myeloperoxidase (MPO)	Induced Sputum	Airway neutrophilic activation, degranulation, and oxidative damage.	Surrogate marker for quantifying the localized neutrophilic burden in the airways [6].
Metabolomic Markers	Sputum Supernatant	Localized oxidative stress, altered cellular metabolism, and disrupted mucus hydration.	Experimental tools utilized for deep molecular endotyping and discovering novel therapeutic targets [7].
H_2_O_2_ and 8-Isoprostane	EBC/Sputum/Urine	Lipid peroxidation and generalized oxidative stress in the lungs.	Quantifies the total oxidative damage burden in COPD patients [29].
Fractional Exhaled Nitric Oxide (FeNO)	Exhaled Breath	Nitric oxide synthesis driven by localized airway epithelial inflammation.	Aids in identifying asthma-COPD overlap or specifically detecting mixed inflammatory profiles responsive to steroids [30].
Extracellular Vesicles	Peripheral Blood	Endothelial activation, cellular apoptosis, and structural tissue damage.	Emerging diagnostic markers being investigated for systemic, cardiovascular, and vascular involvement in COPD [30].
Quantitative CT scans Imaging Parameters	Lung imaging	Non-invasive structural analysis	Assesses airway remodelling and air trapping; screens for concurrent lung cancer risk [30].

**Table 3:** Cellular and Molecular Biomarkers in Interstitial Lung Disease (ILD). * MMP-7 cutoff > 5.0 ng/mL (prognostic threshold). Other biomarker cutoffs are assay dependent.

Biomarker	Primary Biological Source	Pathophysiological Association	Clinical Utility
Krebs von den Lungen-6 (KL-6)	Peripheral Blood (Serum)/BAL	Synthesized by alveolar type II epithelial cells; elevated levels reflect ongoing epithelial cell injury, hyperplasia, and active fibrogenesis.	A widely used diagnostic and prognostic marker; it predicts disease severity, accelerated lung function decline, and the risk of acute exacerbations and poor outcomes [40].
Surfactant Proteins A & D (SP-A & SP-D)	Peripheral Blood (Serum)/BAL	Secreted by alveolar type II cells; increased systemic levels indicate disruption of the alveolar–capillary barrier and epithelial hyperplasia due to lung injury	Useful for assessing the degree of lung impairment, monitoring the clinical course, and predicting long-term survival in both IPF and autoimmune-related ILDs [40].
Matrix Metalloproteinase-7 (MMP-7) *	Peripheral Blood (Plasma/Serum)	Reflects excessive extracellular matrix remodelling and active tissue fibrogenesis.	Highly validated as a diagnostic and prognostic marker; helps differentiate IPF from healthy controls and alternative ILDs, and strongly predicts disease progression and mortality [44].
C-C Motif Chemokine Ligand 18 (CCL 18)	Peripheral Blood (Serum)	Indicates M2 polarization of alveolar macrophages, which secrete profibrotic mediators driving interstitial remodelling.	A robust predictor of disease progression and increased mortality risk, particularly useful in cohorts with systemic sclerosis-associated ILD (SSc-ILD) and IPF [42,44].
C-X-C Motif Chemokine Ligand 4(CXCL4)	Peripheral Blood (Serum)	Chemokine signalling associated with endothelial dysfunction and fibrotic progression.	Serves as a potential biomarker for identifying progressive fibrosis and worsening disease trajectories, notably in SSc-ILD [42].
Autoantibodies (e.g., Anti-Scl-70, Anti-MDA5)	Peripheral Blood (Serum)	Systemic autoimmunity driving localized, potentially aggressive lung inflammation and fibrosis.	Crucial for diagnosing underlying Connective Tissue Disease-associated ILDs (CTD-ILD); highly predictive of an aggressive clinical course and faster pulmonary function decline [41].
Chitinase-3-like protein 1 (YKL-40)	Peripheral Blood (Serum)	Associated with active tissue remodelling and the activation of macrophages and epithelial cells.	An emerging candidate biomarker currently being investigated for its utility in tracking the progression of fibrosing ILDs [47].

**Table 4:** Cellular and Molecular Biomarkers in Lung cancer. * PD-L1 cutoff ≥ 50% (High expression/ICI eligible). Other biomarker cutoffs are assay dependent.

Biomarker	Primary Biological Source	Pathophysiological Association	Clinical Utility
Epidermal Growth Factor Receptor (EGFR)	Tumour Tissue/Liquid Biopsy (Plasma)	Activating mutations (e.g., Exon 19 deletions, Exon 21 L858R) drive uncontrolled epithelial cell proliferation and cancer growth	Highly predictive driver mutation; used to select patients for targeted EGFR tyrosine kinase inhibitors (TKIs) like Osimertinib in early and advanced stages [58].
Anaplastic Lymphoma Kinase (ALK)	Tumour Tissue/Liquid Biopsy	Chromosomal rearrangements or fusions (e.g., EML4-ALK) lead to constitutive kinase activity promoting oncogenesis.	Identifies patients eligible for highly effective targeted ALK inhibitors (e.g., alectinib, crizotinib) [58].
ROS1	Tumour Tissue/Liquid Biopsy	Gene fusions resulting in the continuous activation of the ROS1 signalling pathway, driving tumour progression	Predictive biomarker to determine eligibility for targeted therapies such as entrectinib or crizotinib [58].
Kirsten rat sarcoma viral oncogene homolog (KRAS (specifically G12C))	Tumour Tissue/Liquid Biopsy	Point mutations cause the KRAS protein to remain in an active state, driving continuous cellular division; frequently co-mutated with TP53 or STK11	Identifies candidates for direct KRAS G12C inhibitors (e.g., sotorasib, adagrasib) in advanced NSCLC [58].
MET (Exon 14 skipping & Amplification)	Tumour Tissue/Liquid Biopsy	Aberrations cause decreased receptor degradation or receptor overexpression, sustaining oncogenic signalling	Used to guide treatment with MET-specific inhibitors (e.g., capmatinib, tepotinib) [58].
BRAF (V600E)	Tumour Tissue/Liquid Biopsy	Mutations in the MAPK/ERK pathway result in aggressive cellular growth and proliferation	Directs the use of combined BRAF and MEK inhibitor therapies (e.g., dabrafenib plus trametinib) [58].
RET & NTRK Fusions	Tumour Tissue/Liquid Biopsy	Rare gene fusions that create hyperactive chimeric kinases driving tumour growth	Determines eligibility for highly specific targeted agents (RET inhibitors like selpercatinib; TRK inhibitors like Larotrectinib) [58].
Programmed Death-Ligand 1 (PD-L1) *	Tumour Tissue (Immunohistochemistry)	Expressed on tumour cells to interact with PD-1 on T-cells, effectively allowing the tumour to evade the body’s immune system	Primary predictive biomarker for immunotherapy; high expression indicates a strong likelihood of response to immune checkpoint inhibitors (e.g., pembrolizumab) [58].
Tumour Mutational Burden (TMB)	Tumour Tissue/Blood	Reflects the total number of mutations within a tumour genome; higher TMB generates more neoantigens, making the tumour more recognizable to the immune system	An emerging predictive biomarker indicating potential responsiveness to immunotherapies, regardless of PD-L1 status [58].
Thyroid Transcription Factor 1(TTF1) & Napsin A	Tumour Tissue (Immunohistochemistry)	Proteins highly expressed in the terminal respiratory unit cells	Crucial diagnostic markers used to definitively subtype NSCLC as Adenocarcinoma (ADC) [58].
p40/p63	Tumour Tissue (Immunohistochemistry)	Markers associated with basal and squamous epithelial differentiation	Diagnostic markers used to accurately differentiate Squamous Cell Carcinoma (SQC) from other lung cancer subtypes [59].
Cytokeratin 19 Fragment (CYFRA 21-1)	Peripheral Blood (Serum)	A soluble fragment of cytokeratin 19 released during tumour cell necrosis and apoptosis, often elevated in epithelial tumours	A widely used diagnostic and prognostic blood biomarker, particularly helpful in monitoring disease progression and treatment response in NSCLC [58].
Carcinoembryonic Antigen (CEA)	Peripheral Blood (Serum)	An oncofoetal glycoprotein frequently overexpressed in adenocarcinomas	Used alongside other serum markers to monitor lung cancer recurrence and evaluate long-term prognosis [58].
Neuron-Specific Enolase (NSE) & ProGRP	Peripheral Blood (Serum)	Enzymes and peptides associated with neuroendocrine differentiation	Clinically utilized to aid in the diagnosis, staging, and continuous monitoring of Small Cell Lung Cancer (SCLC) [59].

## Data Availability

No new data were created or analyzed in this study. Data sharing is not applicable to this article.
